# Extensive Evaluation of the Overall Workplace Experience and Job Satisfaction of Dentists in Saudi Arabia

**DOI:** 10.1155/2022/4968489

**Published:** 2022-01-07

**Authors:** Ali A. Assiry, Alanod Alnemari, Abdul Habeeb Adil, Mohmed Isaqali Karobari, Fazlur Rahman Sayed, Anand Marya, Syed Nahid Basheer, Charu Mohan Marya

**Affiliations:** ^1^Preventive Dental Science Department, Faculty of Dentistry, Najran University, Saudi Arabia; ^2^Ministry of Health, Saudi Arabia; ^3^Department of Community Dentistry, School of Dental Sciences, Universiti Sains Malaysia, 16150 Kubang Kerian, Kelantan, Malaysia; ^4^Department of Restorative Dentistry & Endodontics, Faculty of Dentistry, University of Puthisastra, Phnom Penh, Cambodia; ^5^Department of Conservative Dentistry & Endodontics, Saveetha Dental College & Hospitals, Saveetha Institute of Medical and Technical Sciences University, Chennai, 600077 Tamil Nadu, India; ^6^Badr Al Samaa Group of Hospitals, Oman-Al Khuwair Branch, Oman; ^7^Department of Orthodontics, Faculty of Dentistry, University of Puthisastra, Phnom Penh, Cambodia; ^8^Department of Orthodontics, Saveetha Dental College, Saveetha Institute of Medical and Technical Sciences, Saveetha University, Chennai, India; ^9^Department of Restorative Dental Sciences, College of Dentistry, Jazan University, Jazan 45142, Saudi Arabia; ^10^Public Health Dentistry, Sudha Rustagi College of Dental Sciences, Faridabad, India

## Abstract

**Background:**

Working conditions, job satisfaction, and their overall impact on a dentist's job satisfaction are critical for future employment and retaining of dentists.

**Objectives:**

This study is aimed at determining the factors influencing the job satisfaction level among dentists. It is also aimed at evaluating how personal (age, gender) and professional (type, type of qualification, and year of practice) characteristics influence overall job satisfaction.

**Methods:**

For data collection, a structured self-administered questionnaire was used, in which one part collected information on personal characteristics. At the same time, the other contained a questionnaire related to job satisfaction. The German validated version of the questionnaire had a 10-point Warr-Cook-Wall (WCW) scale developed by Warr et al. in 1979. Each item was rated on a 5-point Likert scale, with 5 representing excessive satisfaction and 1 representing extreme dissatisfaction.

**Results:**

The results revealed that dentists in Saudi Arabia have a higher satisfaction level with “colleagues and fellow workers” (26.5%). The relation between the years of practice was highly significant. However, they were dissatisfied with their “income” (22.6%), and when compared with concerning age, gender, profession, and their practice years, this finding was highly significant.

**Conclusion:**

A higher percentage of satisfaction was seen with the “fellow and colleague's workers” dimension. At the same time, “income” was the aspect with which the dentists showed extreme dissatisfaction.

## 1. Introduction

Attaining life satisfaction is a worldwide ambition. One factor of our emotions affecting our satisfaction levels in life is our approach towards work. Job satisfaction has been defined as “a pleasant or optimistic expressive state developing from the assessment of one's job or work experiences” [1]. Learning job satisfaction is crucial because of its stated effect on a person's mental and physical wellbeing and its likely effects on job-related performance and behaviors [2]. In dentistry, job satisfaction has been related to numerous health system outcomes, patient care and job presentation, and overall living satisfaction [3]. Therefore, job satisfaction is not a solo object but a compound set of interrelationships between roles, tasks, interactions, responsibilities, rewards, and incentives [4]. Dentistry is an occupation where professionals are subjected to many job-related factors affecting their motivation and overall wellbeing. Several investigations have reported a high prevalence of psychological and physical disorders in dental practice [5].

Job satisfaction is another aspect in any field, including dentistry which also needs to be dealt with to improve the overall performance of dentists in the field. According to a study done by Spector in 1997, it is defined as how an individual experiences their jobs, several aspects of their occupation, and the degree to which they like or dislike their jobs [6]. Numerous factors at work—dentists' position in society, self-realization, social recognition, and many other aspects every day—give and enhance job satisfaction [5]. Fulfillment of needs results in some reward, which can be either intrinsic or extrinsic. The former is derived from the employee, e.g., taking pride and feeling good about a job well done, whereas the latter pertains to rewards given by another person [7]. The intrinsic inspirational factors are responsibility, recognition, and work tasks while the extrinsic factors include working conditions, salary, and job security [8]. For dentists, both the intrinsic and extrinsic factors are essential, but intrinsic factors have the most significant positive impact on job satisfaction [9]. Job satisfaction problems have often surfaced in prominent issues such as an exceedingly high turnover rate of dentists, which often manifests as loss of productivity [10]. Many experts have attributed high turnover rates and productivity loss to market behavior, impacting workplace productivity, effort, absenteeism, and employee turnover [6].

Job motivation and satisfaction are emotional responses to existing workplace conditions and directly impact the ability of individuals to pursue goals and perform. However, job satisfaction and motivation can go a long way in helping increase work performance. Healthcare organizations can implement several steps to enhance job satisfaction, principally by focusing on the individual and collective interests of the existing and future staff [11]. This job enthusiasm helps to increase the patient care from which the entire dental care system benefits [5].

For example, a country such as India has approximately 301 dental colleges, with around 27,000 graduates passing out each year. When the dentists and their availability are equated to this vast population, the supply and demand ratio is far insufficient and inadequate [12]. In India, the dentist : population ratio is 1 : 10,000. However, the actual ratio in rural India is much higher; i.e., one dentist is serving over a population of 2,50,000. For the 72% of rural population residing in India, there are only 2% dentists available [13–17].

When matched with the internal expectations of employees, income and appreciation have a significant role to play when it comes to overall job satisfaction. The economic changes, shortages in auxiliary staff, and changing workplace structures contribute to the variations experienced in the dental office and have inferences for active clinical practice [10]. The working circumstances and their influences on the job satisfaction of dentists must be considered when recruiting and retaining dentists. Therefore, we must make a conscious effort to understand the work environment and associated factors that directly impact the job satisfaction levels of dentists. There are very few studies regarding job satisfaction among dentists, which is why the present study was carried out among dentists working in the dental field across Saudi Arabia regarding their job satisfaction. The study was aimed at assessing the factors (extrinsic-hygiene and intrinsic-motivation) influencing dentists' overall job satisfaction level in Saudi Arabia. It was also aimed at evaluating how personal (age, gender) and professional (type, type of qualification, and year of practice) characteristics have influenced overall job satisfaction. Based on results obtained from the study, there are recommendations on issues impacting dentists' overall recruitment and retention.

## 2. Materials and Methods

### 2.1. Location of Study, Design, and Ethical Approval

The present study is of a cross-sectional design on the job satisfaction of dentists in Saudi Arabia. The study was assigned and approved by the ethical committee at the Faculty of Dentistry, Najran University, with the ethical approval number 2021/0013.

### 2.2. Questionnaire

A structured self-administered questionnaire was prepared and provided to the participants for data collection. It consisted of two sections. The first part collected information on personal characteristics such as age, gender, profession, type of practice, and years of practice. The second part comprised a questionnaire on job satisfaction. The German validated version of the questionnaire had a 10-point Warr-Cook-Wall (WCW) scale developed by Warr et al. in 1979. Each item was rated on a 5-point Likert scale, with 5 representing excessive satisfaction and 1 representing extreme dissatisfaction. The data was collected from participants from the 1^st^ of January 2021 to the 30^th^ of April 2021.

### 2.3. Inclusion and Exclusion Criteria

- Dentists with at least 1 year of work experience were included in the study
- Dentists willing to participate in the study were included

### 2.4. Sample Size

The data was collected over four weeks. The total sample size for the study was 155 registered dentists. The researchers personally did the collection and distribution of all questionnaires. The anonymity of the questionnaire was ensured.

### 2.5. Data Analysis

The statistical analyses were done using SPSS (Statistical Package for the Social Sciences) version 26. Means and standard deviations were used to present the continuous data. The frequency counts and percentages were utilized to summarize the categorical data.

Further, multiple linear regression analysis was carried out to construct models for job satisfaction using individual characteristics (age, gender, profession, type of practice, years of academic practice, and years of private practice). Finally, binary logistic regression analysis was done to determine the contribution of various individual characteristics to job satisfaction. The cut-off level for statistical significance was taken at 0.05.

## 3. Results

**Figure 1:**
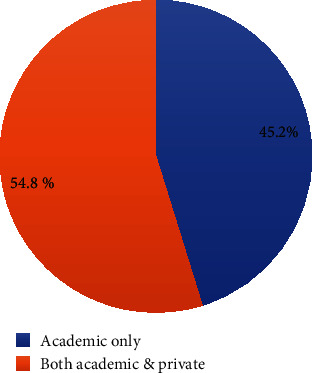
Percentage distribution of dentists according to the type of practice.

**Figure 2:**
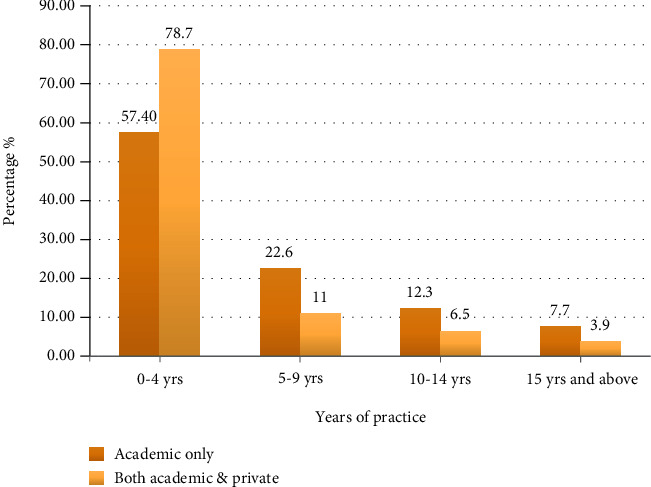
Percentage distribution of dentists according to years of practice.

The mean age of dentists was 33.20 ± 6.7 years, and female dentists exceeded the male dentists by 5%; specialist dentists were 3 times more in number than general dentists (Table 1). The type of practice is shown in Figure 1. Dentists with lesser experience were more involved in private practice and academics, whereas the dentists with higher experience in the field preferred only academics (Figure 2).

Table 2 shows the percentage distribution of dentists' satisfaction levels for different domains of the Job Satisfaction Scale. The higher percentage of satisfaction with the job was with the “colleagues and fellow workers” dimension, with 26.5% of dentists showing extreme satisfaction. “Income” was the aspect with which the dentists showed extreme dissatisfaction (22.6%).

Each aspect of job satisfaction was rated on a 5-point Likert scale, with 5 representing excessive satisfaction and 1 representing extreme dissatisfaction. For the sake of analysis, the Likert scale was dichotomized by combining scores 1 and 2 (extreme dissatisfaction and dissatisfaction) as “not satisfied” and scores 3, 4, and 5 (partial satisfaction, satisfaction, and extreme satisfaction) was counted as “satisfied.” The scores of this scale ranged from 0 to 9 (excluding overall job satisfaction domain) with a mean score of 7.59 ± 2.25 for this present study. The score of 4 and below was considered “low satisfaction,” and 5 and above was considered “higher satisfaction.”

Table 3 shows the comparison of personal characteristics, including age, gender, profession, type of practice, and years of practice, with overall job satisfaction. The analysis showed statistically significant difference (*p* < 0.05) in age which were attributed to “income” and “opportunity to use abilities”; gender was attributed to “income” and “amount of variety in the job”; profession was attributed to “income,” “amount of working hours,” and “amount of job variety”; type of practice was attributed to “working method freedom “ and “hours of work”; years of practice in academic institution was attributed to “income,” whereas years of practice in private was attributed to “freedom of working method” and “fellow workers and colleagues”.

Table 4 shows the influence of individual characteristics on overall job satisfaction, and the multiple linear regression analysis was carried out to assess the impact of the 6 independent variables on job satisfaction. Model 3 with age, gender, and profession explained for 49% of the variation for job satisfaction and was significantly associated with job satisfaction. Model 4 with age, gender, profession, and type of practice was significantly related to job satisfaction. Binary logistic regression analysis was done to determine the contribution of various individual characteristics towards job satisfaction. The result showed that specialist dentists were 3.55 times more satisfied than general dentists (95% CI 1.18 to 10.66) (Table 5).

## 4. Discussion

Our study assessed personal characteristics that included age, gender, profession, type of practice, and years of practice; job satisfaction; and the association between the two. The mean age of dentists was 33.20 ± 6.7 years, in which 62.6% (*n* = 97) were below 33 years of age, whereas 37.4% (*n* = 58) were above 33 years of age. Similar results were found in the study done by Luzzi et al., where most respondents belonged to a younger age group. In the present study, female dentists (52.3%) exceeded the male dentists (47.7%) by 5%. The male and female dentists varied significantly, with female dentists showing high scores on this aspect. The gender differences emphasized that women are more selecting dentistry as a career as compared with males. In our study, M.D.S./specialist dentists (76.1%) were 3 times more in number than general dentists, which contrasted with the study done by Puriene et al. in which 17.4% were specialist dentists.

When responders were asked about the type of study, 54.8% had their private practice and served in institutions as academicians while 45.2% were solely academicians. Luzzi et al. stratified the dental practice into public and private practice in which a majority of the responders belonged to public practice.

### 4.1. Comparing Demographic Details with Overall Job Satisfaction

This study results revealed that dentists in Saudi Arabia have a higher satisfaction level with “fellow workers and colleagues” (26.5%). The relation between this domain and the personal characteristic, i.e., years of practice in the private sector, was highly significant. This significant association between satisfaction and work colleagues is related to cordial relationships among them. However, they were dissatisfied with their “income” (22.6%), and when compared with age, gender, profession, and years of practice in academic institutions, income was highly significant. A significant association was found because the responders feel the income in an academic institution is much less for the faculty compared to the number of years spent studying the course and the enormous financial expenses involved. Also, this could be attributed to the fact that the profession of doctors is highly respected, and hence, they feel income is not apt for the profession.

The domain “freedom of working method” was found to be highly significant with “type of practice” and “years of practice in private.” This could be because general dentists can perform all treatment procedures while specialists can perform treatments related to their respective fields. In private practice, as it is one's own setup, the dentist is entirely in charge and has the full freedom of working in the clinic. In a study done by Luzzi et al., dissatisfaction with academic practice was endorsed to important factors linking to administration intrusion, career pathway affairs, and job interference into family life.

The domain “hours of work” was found to be significantly associated with “profession” and “type of practice.” Similar results were found in the study done by Goetz et al. that dentists who work fewer hours weekly at a young age and work at their practice for many years are more likely to be satisfied with their jobs. Considering the Theory of Two-Factor Job Satisfaction, income or working hours are considered external-hygiene factors that contribute to undesirable emotions at the workplace.

This demonstrates how their deficiency can cause unhappiness. Internal motivators such as recognition, on the other hand, add importance and proceed as a means for optimistic impressions about being a dental surgeon [18]. Dentists who have worked in their own practice for a long time are liable to have paid off the dues related to possessing their practices, lowering the financial burden that young dentists are subjected to. It has been demonstrated that the number of working years of experience positively influences self-reported job satisfaction [19]. Furthermore, an undesirable relationship was discovered between increased working hours, work engagement, and rising age [20]. According to the study, job satisfaction and various work attributes have a solid and substantial relationship with age due to different job expectations or adapted working standards during the separate stages of a dentist's profession [21]. Generally, the qualities of the part that cause high and low job satisfaction for dentists may differ and depend on age [9].

The present study presented the mean overall job satisfaction score of 7.59 ± 2.25 out of 9. It represents that overall job satisfaction surpasses “being satisfied” and methods to “being very satisfied” with dental practice. A previous survey from Lithuanian dentists reported the mean score of overall job satisfaction to be remarkably high, i.e., 4.06 out of 5 from general practitioners of California; the mean score of overall job satisfaction was 63 out of 100; from dentists of South Korean, it was 3.2 out of 5. A survey on Canadian orthodontists showed a comparable low job satisfaction level with a mean value of 4.0 out of 5. The level of job satisfaction among dentists of Lithuania is exceedingly high when associated with other medical specialties. For the doctors who were working at the primary health care organizations in Lithuania, the job satisfaction score was 4.74 on a 7-point scale, while the job satisfaction level between all staff fellows at an Estonian hospital was 3.86 out of 5.

In the present study, stagewise multiple linear regression analysis was done to investigate further the association between dentists' overall job satisfaction in Saudi Arabia as dependent variable and job satisfier factors as independent variables or predictors. The purpose was to ascertain the contribution of each job satisfier factor to the variance in overall job satisfaction. The *R*-square indicates how much variance in overall job satisfaction is present. Altogether, age, gender, and profession (Model 3) and age, gender, profession, and type of practice (Model 4) showed a statistically significant correlation with job satisfaction with a *p* value < 0.05 where profession alone contributed to 49% of the variation.

In binary logistic regression analysis, comparing the contribution of each of the job satisfier factors, the factor, “profession” with the most significant beta coefficient (3.55), when the variance explained by all other job satisfaction factors in the model was controlled for, made the highest unique contribution to explaining overall job satisfaction. As shown in Table 5, the other factors (age, gender, type of practice, years of practice academic, and years of practice private) were less significant. So, we can conclude that specialist dentists were 3.55 times more satisfied than general dentists with a 95% confidence interval of 1.18 to 10.66. Overall, our findings compare favorably with a previous study on primary care physicians' job satisfaction, which also uses a WCW instrument to evaluate job satisfaction [22].

How a dentist thinks about himself as a professional and how he perceives his job remain critical and fundamental to how they practice. If the dentist is unhappy, it certainly concerns his practice. This study's outcome shows the dental profession's disputes that claim courtesy to enhance the job satisfaction level among dentists in Saudi Arabia that would invariably help improve the entire dental care system.

## 5. Limitations

The total sample size for this study was 155, which could have been a limitation, but it was specified beforehand that only completed responses would be accepted. The study was of a cross-sectional design, and convenience sampling was used for this study. The responses were gathered by sending reminders, but previous literature has shown that health professionals usually offer fewer questionnaire surveys. Also, the data were obtained from different regions to ensure the generalizability of the study's findings.

## 6. Recommendations

According to the theory of job satisfaction, two factors play vital roles in influencing satisfaction in one's job: external factors such as working hours and income all contribute to unpleasant thoughts at the workplace. This demonstrates how their nonexistence can cause dissatisfaction. Instead, internal inspirations such as identification add value and catalyze optimistic thoughts about working as a dental surgeon. To achieve the desired level of satisfaction in dentistry, the following pride system could be implemented to improve the quality of work in dentistry. Future studies can also include the use of Job-Related Tension Index which utilizes 11 items to determine the frequency of stress-laden occurrences at work along with the extent to which a professional role is overloaded.

### 6.1. The Pride System [23]

- Provide an optimistic working atmosphere
- Reward and appreciation
- Involve and enhance employee meetings
- Develop the skills and abilities of your workforce
- Evaluate and assess job satisfaction

Along with the pride system, a standard salary, apt for the designation and workload, must be implemented throughout the country. Also, since this was a pilot study aimed at a specific population, it would be recommended to conduct a similar study based on multicultural populations to be able to gauge the general levels of job satisfaction and try and bring up the standards of dentistry in terms of job satisfaction on a worldwide scale.

## 7. Conclusion

According to the Theory of Two-Factor Job Satisfaction, both extrinsic and intrinsic inspiring factors are essential for dentists, but intrinsic inspiring factors positively influence job satisfaction. Finally, our study demonstrates that dentists in Saudi Arabia are satisfied with their work. Of the 5 extrinsic and 4 intrinsic factors (freedom of working method, physical working conditions, fellow workers and colleagues, amount of responsibility, recognition for work, income, hours of work, variety in job, and opportunity to use abilities) influencing overall job satisfaction, it was found that a higher percentage of satisfaction with their job was with the “fellow workers and colleagues” aspect and “income” was the aspect with which the dentists showed extreme dissatisfaction. The result showed that specialist dentists were more satisfied than general dentists. The findings of this study will be helpful in future activities to improve dentists' working conditions and ensure the quality of dental care. Dentists require assistance from ongoing professional training programs and a helpful team to put their skills to practice.

## Figures and Tables

**Table 1:** Percentage distribution of dentists according to age, gender, and profession.

	Frequency (*n*)	Percent (%)
*Age (years)*		
Less than 33	97	62.6
More than 33	58	37.4
*Gender*		
Male	74	47.7
Female	81	52.3
*Professional qualification*		
General dentists	37	23.9
Specialist dentists	118	76.1
Total	155	100.0

**Table 2:** Distribution of dentist satisfaction level in percentage within different domains of the scale.

Sl. no.	Questions	Percentage distribution on 5-point Likert scale									
		Extreme dissatisfaction		Dissatisfaction		Partial satisfaction		Satisfaction		Extreme satisfaction	
		*N*	%	*N*	%	*N*	%	*N*	%	*N*	%
1	Physical working condition	6	3.9	16	10.3	81	52.3	35	22.6	17	11.0
2	Freedom of working method	8	5.2	20	12.9	57	36.8	54	34.8	16	10.3
3	Colleagues and fellow workers	3	1.9	7	4.5	34	21.9	70	45.2	41	26.5
4	Recognition for work	15	9.7	27	17.4	47	30.3	57	36.8	9	5.8
5	Amount of responsibility	8	5.2	14	9	61	39.4	58	37.4	14	9
6	Income	35	22.6	29	18.7	48	31	38	24.5	5	3.2
7	Opportunity to use abilities	8	5.2	42	27.1	66	42.6	35	22.6	4	2.6
8	Hours of work	10	6.5	32	20.6	62	40	49	31.6	2	1.3
9	Amount of variety in job	8	5.2	45	29	75	48.4	22	14.2	5	3.2
10	Overall job satisfaction	7	4.5	31	20	57	36.8	54	34.8	6	3.9

**Table 3:** Comparison of individual characteristics with overall job satisfaction.

Sl no.	Questions	Age		Gender		Profession		Type of practice		Years of practice in academic institution		Years of practice in private	
		Chi-square value	*p* value	Chi-square value	*p* value	Chi-square value	*p* value	Chi-square value	*p* value	Chi-square value	*p* value	Chi-square value	*p* value
1	Physical working condition	3.211	0.073	1.324	0.250	0.891	0.345	0.187	0.665	2.043	0.153	3.058	0.080
2	Freedom of working method	0.432	0.511	3.749	0.053	0.680	0.410	7.772	0.005^∗∗^	1.757	0.185	12.352	≤0.001^∗∗∗^
3	Colleagues and fellow workers	2.328	0.127	0.644	0.422	0.088	0.767	2.733	0.098	3.765	0.052	8.328	0.004^∗∗^
4	Recognition for work	0.411	0.522	0.118	0.731	0.189	0.664	0.124	0.725	0.045	0.832	0.045	0.832
5	Amount of responsibility	1.127	0.288	0.054	0.817	2.202	0.138	0.001	0.976	1.066	0.302	0.071	0.790
6	Income	11.24	≤0.001^∗∗∗^	7.807	0.005^∗∗^	27.578	≤0.001^∗∗∗^	2.792	0.095	7.118	0.008^∗∗^	0.507	0.477
7	Opportunity to use abilities	4.989	0.026^∗^	0.002	0.965	0.001	0.979	0.240	0.624	1.114	0.291	1.414	0.234
8	Hours of work	1.029	0.310	0.551	0.458	4.447	0.035^∗^	6.402	0.011^∗^	0.971	0.325	0.034	0.854
9	Amount of variety in job	0.085	0.771	4.566	0.033^∗^	6.359	0.012^∗^	1.087	0.297	0.577	0.448	0.709	0.400
10	Overall job satisfaction	4.056	0.044^∗^	5.271	0.022^∗^	9.211	0.002^∗∗^	1.134	0.287	1.694	0.193	0.273	0.602

^∗^ *p* value < 0.05, ^∗∗^*p* value < 0.01, and ^∗∗∗^*p* value < 0.001.

**Table 4:** Multiple linear regression model for job satisfaction.

	*R*	*R* ^2^	*R* ^2^ change	*p* value	*F* value
1	0.113	0.013	—	0.16	1.99
2	0.128	0.016	0.003	0.28	1.26
3	0.226	0.51	0.49	0.04^∗^	2.72
4	0.244	0.59	0.08	0.05^∗^	2.36
5	0.244	0.59	0	0.10	1.88
6	0.250	0.63	0.04	0.13	1.64

^∗^Statistical significances of difference: ≤0.05. (1) Model 1: age. (2) Model 2: age and gender. (3) Model 3: age, gender, and profession. (4) Model 4: age, gender, profession, and type of practice. (5) Model 5: age, gender, profession, type of practice, and years of practice academic. (6) Model 6: age, gender, profession, type of practice, years of practice academic, and years of practice private.

**Table 5:** Binary logistic regression analysis with job satisfaction as dependent variable.

Factor	*p* value	Exp(*B*) odds ratio	95.0% C.I. for Exp(*B*)	
			Lower	Upper
Age	0.93	1.00	0.82	1.22
Gender	0.59	0.75	0.26	2.15
Profession	0.02^∗^	3.55	1.18	10.66
Type of practice	0.41	0.64	0.22	1.84
Years of practice academic	0.87	1.01	0.82	1.25
Years of practice private	0.27	0.93	0.83	1.05

^∗^Statistical significances of difference: ≤0.05.

## Data Availability

All data used for backing the outcome of this study are comprised within the article.
